# Direct Intracranial Extension of Malignant Eccrine Spiradenoma of the Face

**DOI:** 10.4021/jocmr2009.07.1249

**Published:** 2009-08-20

**Authors:** Srinivas B. Pedamallu, Justin Murphy, Douglas Boyd, Dominic Martin-Hirsch, Khaled Al-Zwae

**Affiliations:** a ENT, Calderdale Royal Hospital, Halifax, United Kingdom, HX3 0PW; b Radiology, Calderdale Royal Hospital, Halifax, United Kingdom, HX3 0PW

## Abstract

Malignant eccrine spiradenomas are exceedingly rare tumours. They are aggressive tumours normally arising in long-standing benign eccrine spiradenomas. We present a case of malignant eccrine spiradenoma of the right side of the face with direct intracranial extension without distant metastasis. A 48 years old woman presented with a large exophytic tumour on the right side of her face. Radiological imaging of the head and neck region revealed extensive invasion of the facial tissues around right orbit, maxilla and extension into the middle cranial fossa involving the right temporal lobe. She underwent craniotomy and debulking of the right temporal lobe and biopsy of the facial tumour. Histopathological findings were consistent with malignant eccrine spiradenoma. This is a rare case of facial malignant eccrine spiradenoma with direct intracranial extension with no distant metastasis. A literature search revealed that it is first case to be reported of this kind.

**Keywords:**

Eccrine carcinoma; Spiradenoma; Adnexal carcinoma; Malignant eccrine spiradenoma

## Introduction

Eccrine spiradenoma is a benign sweat gland tumor that commonly affects young adults. The presentation is often a single nodule that may or may not be tender. In contrast, the malignant eccrine spiradenoma (MES) is an extremely rare tumor, which almost always arises from a pre-existing eccrine spiradenoma. The overall prognosis of malignant eccrine spiradenoma is poor [1].Our case report describes one such aggressive presentation of MES with direct intracranial extension without distant metastasis.

## Case Report

**Figure 1:**
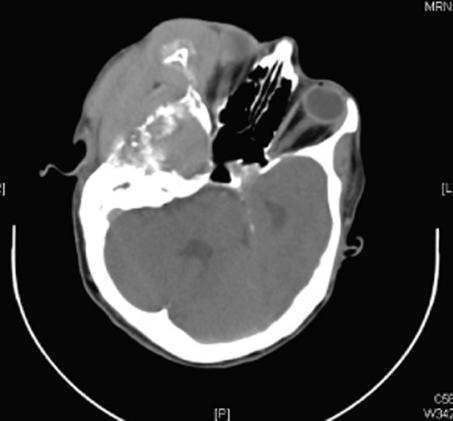
Axial CT scan image showing tumour invading the orbit, right side of skull base and destroying the bones. This is suggestive of aggressive tumour.

A 48 years old lady presented to the emergency department with a large exophytic right-sided facial swelling. She was confused at the time of presentation and examination revealed right-sided facial palsy and chemosis of the right eye. Eye movements and pupillary reactions were normal. Upper and lower limb tone, power, sensation and reflexes were grossly normal. The patient had an emergency CT head which demonstrated a large enhancing tumour with necrotic areas involving the right half of face especially along the right parotid, right infra temporal fossa, right petrous temporal bone, right orbital and maxillary region. The mass extended intra cranially, displacing the right temporal lobe supero-medially causing surrounding brain oedema, left ventricular dilatation and about 1.3 cm of midline shift (Fig. 1). While awaiting fine needle aspiration and further imaging, her consciousness deteriorated further and she was transferred to the care of neurosurgeons. She had emergency craniotomy and debulking of right temporal tumour.

**Figure 2:**
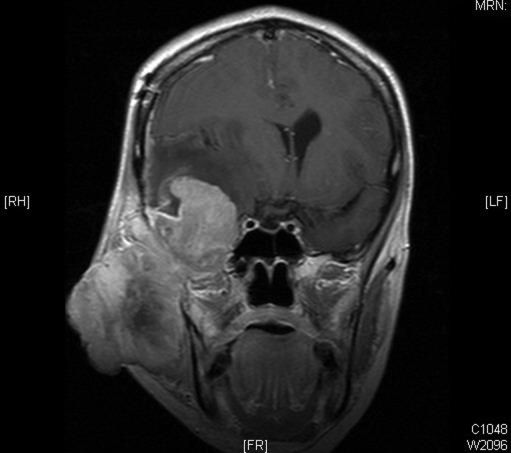
Coronal T1 post contrast MR image showing the tumour extension intracranially into the right temporal lobe causing compression of the right lateral ventricle and midline shift to the left.

Post-operatively, she remained neurologically stable. An MRI scan of the head and neck region demonstrated a large soft tissue mass measuring 10 × 5 cm within the right side of the face extending infero-temporally into the middle cranial fossa with necrotic areas (Fig. 2). The mass was found to be very aggressive and invaded nearby soft tissues, muscles and bones of the orbit,skull base, right ear, right temporomandibular joint, right maxillary sinus, right carotid artery, right jugular vein, right temporal lobe causing cerebral oedema, midline shift and obstructive hydrocephalus. Abnormal enhancing enlarged lymph nodes are noted within the right upper neck particularly level I and II.

Following further assessment, it was decided that the facial lesion was not amenable to curative surgery. Histology of the debulked tumour has shown a malignant eccrine spiradenoma that is histologically highly malignant.

Her case was discussed at the regional multidisciplinary team meeting, where it was decided that she was not a candidate for further surgery or chemo radiotherapy. She was transferred to the palliative care team and died 5 weeks after the diagnosis.

## Discussion

Malignant eccrine spiradinoma (MES) is a rare adnexal carcinoma of the skin. It is normally considered to be a malignant transformation of a pre-existing benign eccrine spriradenoma [1-3]. MES presents at an average age of 59 years (range: 21-92 years) and shows no sex predilection [4]. It tends to preferentially involve the trunk and extremities (92% of reported cases) [4], but there are also published case reports of MES arising in the breast [4], scalp [5, 6], and eyelids [7, 8]. MES has been reported to occur on a traumatized area [9]. Literature review revealed only about 18 reported cases involving the head and neck region [1, 10]. Overall prognosis of malignant eccrine spiradenoma is poor [1].

Primary treatment includes local excision, with or without regional lymphadenectomy [1], with recurrence reported in 17.5% of the cases [4]. Malignant eccrine spiradenoma metastasizes to regional lymph nodes [3], lungs [11], brain, skin [1] , bone [1] and liver [4] (in descending order of frequency). Distant metastases of MES are uncommon even in extensive tumours, such as the one reported here, metastasis are rare but carry a poor prognosis [4]. Radiation therapy alone or in combination with chemotherapy has been used with no benefit in the treatment of patients with metastatic MES [4]. Sridhar et al reported symptomatic improvement and shrinkage of the tumor with tamoxifen therapy in a patient with estrogen receptor-positive eccrine adenocarcinoma [12]. However, the role of hormonal therapy still remains to be determined. Close follow-up of these patients for early detection of recurrence and metastases cannot be overemphasized.

We report the first case of malignant eccrine spiradenoma of the face extending infero-temporally into the middle cranial fossa and involving right temporal lobe. This tumour was locally aggressive, invading bones of the orbit, skull base, right ear, right temporomandibular joint, right maxillary sinus, right carotid artery, right jugular vein and surrounding soft tissues, but had not metastasised to distant sites.

This case report describes one such aggressive presentation of malignant eccrine spiradenoma with intracranial extension with no distant metastasis that unfortunately resulted in the patient's death 5 weeks after the initial diagnosis.
